# Experimental Evaluation of Ocular Rigidity and Pressure-Volume Relationship After Ex-Vivo Scleral Cross-Linking With Riboflavin and Ultraviolet A in Porcine Eyes

**DOI:** 10.7759/cureus.75667

**Published:** 2024-12-13

**Authors:** Nektarios E Klados, Emmanouil Modatsos, Aristotelis Karamaounas, Ioannis Pallikaris

**Affiliations:** 1 Ophthalmology, Medical School, Institute of Vision and Optics, University of Crete, Heraklion, GRC; 2 Ophthalmology, 417 Army Equity Fund Hospital, Athens, GRC; 3 Ophthalmology, "G. Gennimatas" General Hospital of Athens, Athens, GRC

**Keywords:** experimental study, ex-vivo, fresh porcine eyes, intraocular pressure, ocular rigidity, riboflavin, scleral cross linking, ultraviolet a, volume-pressure relationship

## Abstract

Purpose: Scleral cross-linking (SXL) with ultraviolet A (UVA) and riboflavin has already been used in laboratory studies for scleral stiffness increase as a potential treatment for progressive myopia and scleral ectasia. This study aims to investigate whether the regional application of scleral cross-linking (SXL) with ultraviolet A (UVA) and riboflavin in fresh porcine eye globes affects the ocular rigidity as well as its impact on intraocular pressure after an induced acute increase in the volume of intraocular fluid.

Methods: The study included two groups of fresh porcine eyes: an experimental group (n=20) that underwent scleral cross-linking (SXL) with riboflavin and UVA applied to the posterior sclera and a control group (n=20) that did not receive SXL treatment. Subsequently, a balanced salt solution (volumes 50, 100, 150, and 200 μL) was administered into porcine globes via a syringe, and, at the same time, the intraocular pressure (IOP) was continuously monitored by a pressure sensor that was cannulated to the vitreous chamber. The relationship between volume and pressure was obtained, and the ocular rigidity coefficient (K) was calculated according to Friedenwald's law. Finally, scleral strips were dissected from the globes and were examined macroscopically.

Results: In the control group, the mean IOP observed entails gradual, statistically significant increases for higher volumes. Specifically, the mean IOP at 0 μL equals 10 mmHg (SD=0), whereas at 200 μL the mean IOP equals 33.83 mmHg (SD=4.060). The differences were statistically significant with p-values <0.001 in all cases. Similarly, the observed gradual IOP increases in the SXL group were statistically significant with p < 0.001 in all cases except for the comparison of volume 0 μL measurements to volume 50 μL, where the p-value equaled 0.003. Specifically in the SXL group, the mean IOP at 0 μL equals 10.00 mmHg (SD=0.000), the mean IOP at 50 μL equals 13.31 mmHg (SD=2.011), whereas the mean IOP at 200 μL equals 32.06 mmHg (SD=3.078). At no additional injected volume, the differences between the control and the SXL groups were statistically significant. The analysis regarding ocular rigidity indicated significantly higher scores in the control group (K50=0.00812, SD=0.03) compared to the SXL group (K50=0.00552, SD=0.027), t=2.844; p=0.007. The difference regards measures of volumes 0 to 50 μL, while all other rigidity measures were found to be non-significant. Interestingly, the ocular rigidity coefficient in the SXL-treated group did not show changes with an increase in IOP. The macroscopic appearance of the scleral strips showed a significantly increased stiffness of the SXL scleras against the control ones.

Conclusion: This study showed that stiffened scleras did not induce substantial change in ocular rigidity and significant IOP elevations. Studying the biomechanical ocular response of laboratory scleral crosslinking applications supports the development of next-generation crosslinking procedures that may constitute potential therapeutic options for severe ophthalmic diseases like pathologic myopia.

## Introduction

Myopia is a common yet complex ophthalmic condition. Once regarded as a benign refractive error, it is now recognized that even low levels of myopia are associated with an increased risk of various ocular disorders [1]. Researchers have highlighted a global myopia pandemic, underscoring its widespread prevalence [2,3]. While the exact causes of myopia remain unclear, it is believed to arise from a combination of genetic and environmental factors, making its prevention and management both individualized and challenging [4]. A subset of individuals with myopia develops pathological myopia, characterized by progressive and excessive elongation of the eyeball. This condition is now acknowledged as a significant contributor to vision impairment and blindness on a global scale [5].

Axial elongation in progressively myopic eyes is believed to result from scleral remodeling, which weakens the biomechanical integrity of the scleral matrix and leads to scleral thinning [6]. One potential therapeutic approach to address this issue is the cross-linking of the scleral extracellular matrix (SXL) using riboflavin and ultraviolet A (UVA) light. This process induces the formation of additional crosslinks between collagen molecules, thereby increasing the biomechanical stiffness of the scleral tissue [7-11]. Enhanced scleral stiffness can limit axial elongation, potentially decreasing the risk of complications such as macular edema, chorioretinal atrophy and staphyloma, retinal tears, and detachment [12].

Although the technique of SXL with riboflavin and UVA is not a treatment option in living human eyes yet, results in several both ex vivo and in vivo studies indicate that SXL results in scleral stiffening [13-16]. A recent in vivo study of Rhesus monkey's eyes after scleral cross-linking with riboflavin and UVA found that SXL could strengthen the scleral tissues and maintain stability for 12 months postoperatively [17]. In the same study, they did not demonstrate any statistically significant early changes in IOP, measured using the TonoVet^TM^ rebound tonometer, between the control and cross-linked specimens [18]. Except for these IOP measurements, which are useful when considering the safety of the procedure, to the best of our knowledge, the influence of scleral stiffening after SXL application in ocular rigidity has not been studied yet [19].

This study aimed to investigate the potential impact of scleral cross-linking (SXL) with riboflavin and ultraviolet A (UVA) on ocular rigidity and intraocular pressure (IOP). Using an experimental model with porcine scleras, we assessed how SXL-induced stiffness affects the biomechanical response of the eye by measuring IOP changes and calculating ocular rigidity during controlled saline infusion based on Friedenwald’s equation [20].

## Materials and methods

Eye preparation

The study involved two groups of fresh porcine eyes: an experimental group (n=20) that received scleral cross-linking (SXL) with riboflavin and UVA applied to the posterior sclera and a control group (n=20) that did not undergo SXL treatment. Simple random sampling was followed. Porcine eyes were obtained and tested within 24 hours of slaughter from a local meatpacking company. All of them were preserved in a 4°C moist chamber before treatment. Directly before use, the periocular connective tissue and soft muscular tissue were removed with surgical scissors to expose the underlying sclera. All experiments were performed at room temperature.

SXL treatment

**Figure 1:**
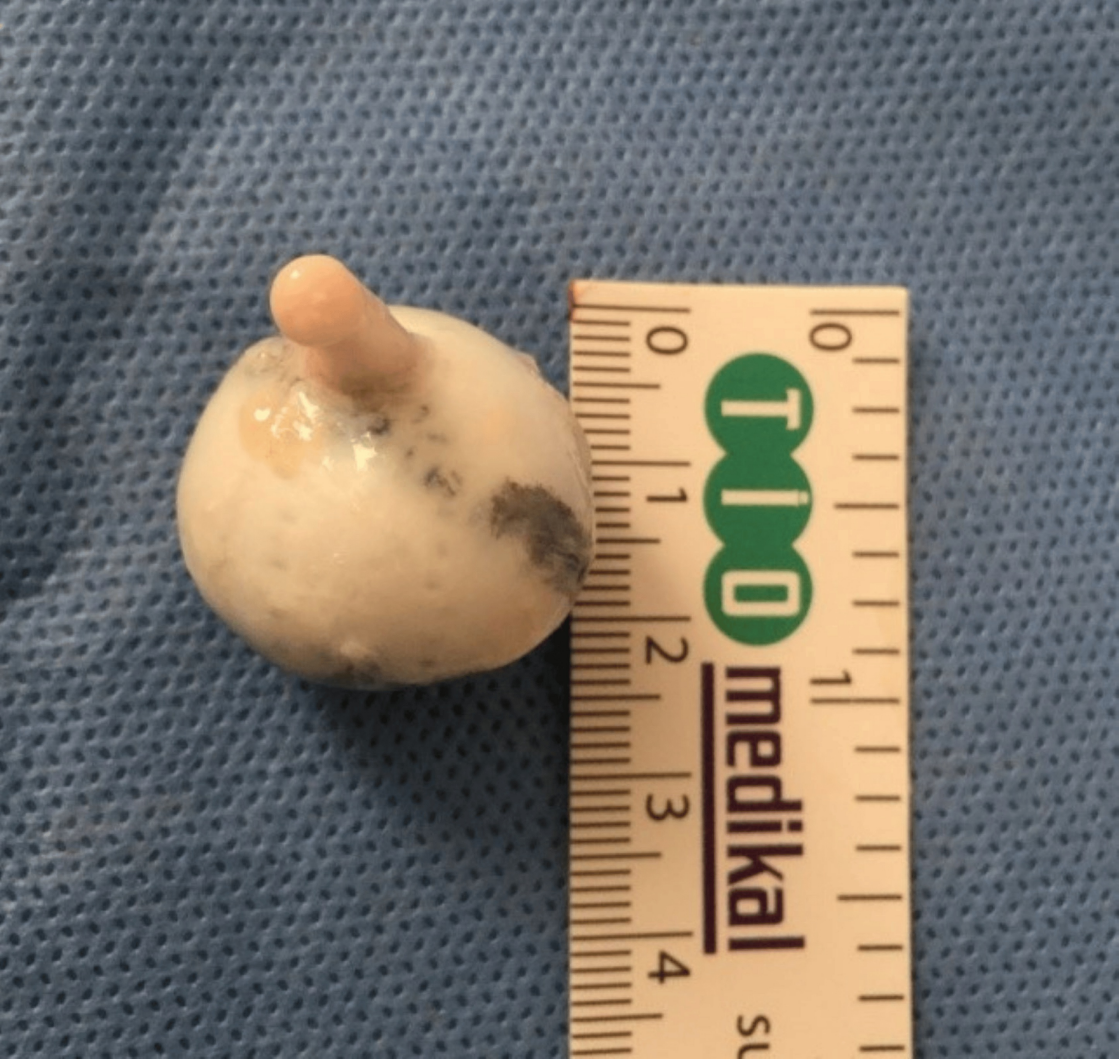
A circular area (D=9mm) of the posterior sclera, simulating the area where the posterior myopic staphyloma is developed, was marked.

In the experimental group (n=20), a circular part of the posterior sclera was initially marked (Figure 1). The treatment area measured 9 mm by 9 mm. It was incubated in riboflavin (0,1% Riboflavin, 20% Dextran 500, Peschke Meditrade GmbH) for 20 minutes to facilitate deep scleral penetration of the riboflavin. Ultraviolet-A irradiation was performed using a commercially available UVA system (UV-X, Peschke Meditrade GmbH). Irradiance was performed for 40 minutes, corresponding to a dose of 3 mW/cm^2^ at a distance of 5 cm from the scleral plane. During treatment, riboflavin solution was applied every 3 to 5 minutes to saturate the sclera. In the control group (n=20), a circular part of the posterior sclera was also marked without receiving SXL treatment. After SXL treatment and marking, eyes from both groups were cannulated in the vitreous cavity for volume injections and pressure measurements.

Measurement procedure

**Figure 2:**
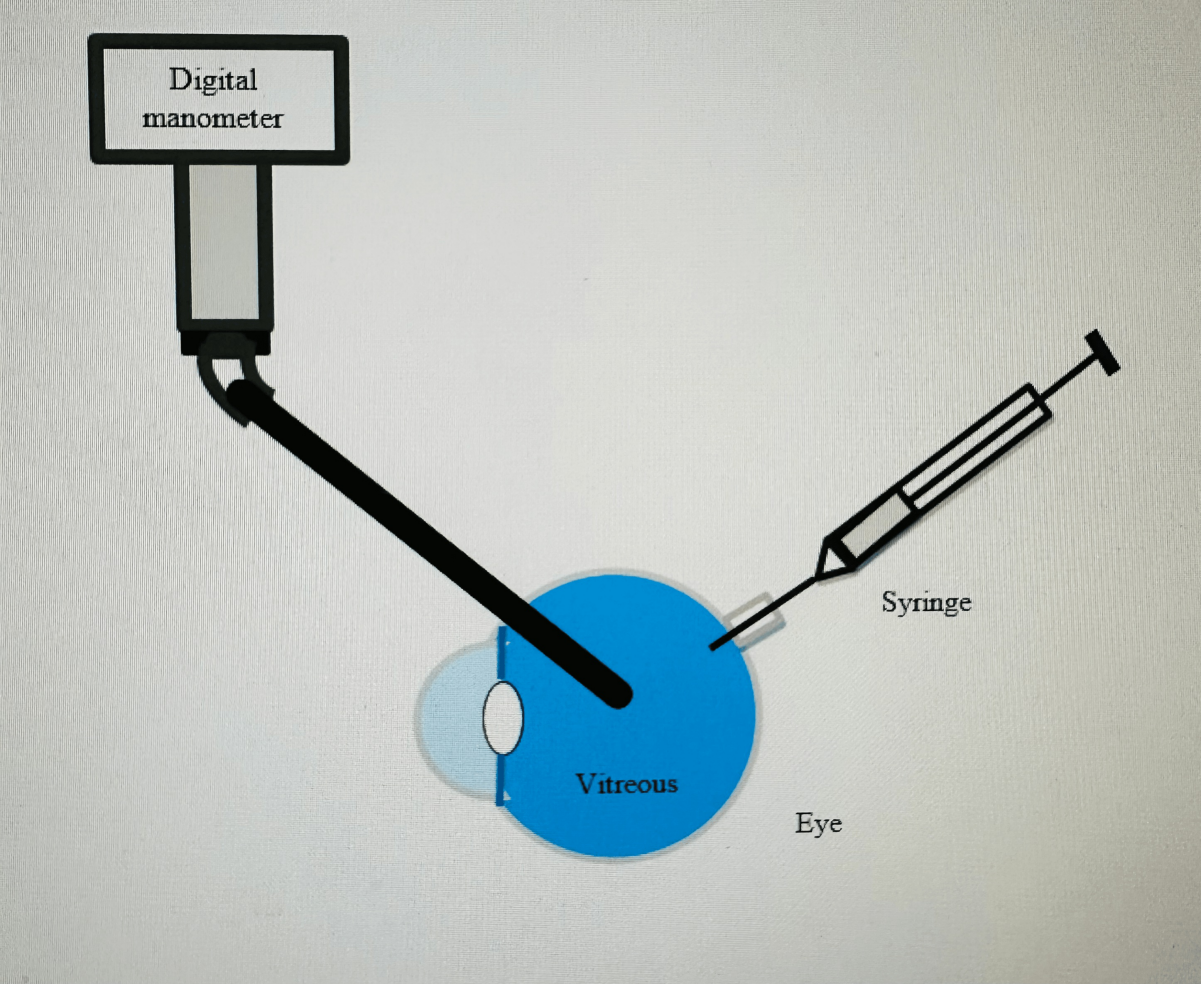
A double cannulation procedure was performed on the examined porcine eye for IOP and volume evaluation. One cannula was attached to a digital manometer to measure the IOP, while the second cannula was connected to an injector for volume assessment.

Eyes were cannulated in the vitreous cavity through two needles; a 25G needle was connected via the optic nerve head to the infusion system with BSS, and another 8G needle was connected to a digital manometer (Figure 2). The initial intraocular pressure (IOP) for all eyes was set to a baseline of 10 mm Hg. A volume of 200 μL of saline was injected into the eye at a rate of approximately 200 μL per minute. IOP was continuously monitored using a pressure sensor, providing real-time measurements during the infusion. The infusion was briefly paused for 20 seconds at specific volumes of 50, 100, 150, and 200 μL to allow the IOP to stabilize and be recorded. The total injection of 200 μL was completed within 2 minutes.

The relationship between the volume of saline injected and the resulting pressure was then analyzed, and ocular rigidity (K) was calculated according to Friedenwald's law. The law stipulates that the ocular rigidity coefficient (K) can be calculated from two measuring points after distention:

$K=\frac{ln(IOP_{2})-ln(IOP_{1})}{V_{2}-V_{1}}$

where IOP_1_, IOP_2_, V_2_>V_1_ are corresponding values of the IOP and intraocular volume V, and ln is the natural logarithm. The dimension of the coefficient (K) is μL^-1^. Afterward, the marked areas of posterior scleral of both the SXL and control groups were excised in order to be examined macroscopically.

Statistical analysis

The IOP scores were expressed with the use of means and standard deviations. A factorial ANOVA was implemented to examine differences in the IOP depending on the scleral group and volume examined. Independent samples t-tests were applied to assess differences between groups in several rigidity measurements. The analysis was carried out using IBM Corp. Released 2019. IBM SPSS Statistics for Windows, Version 26.0. Armonk, NY: IBM Corp., and all statistical significance was set at 0.05 in all cases.

## Results

**Figure 3:**
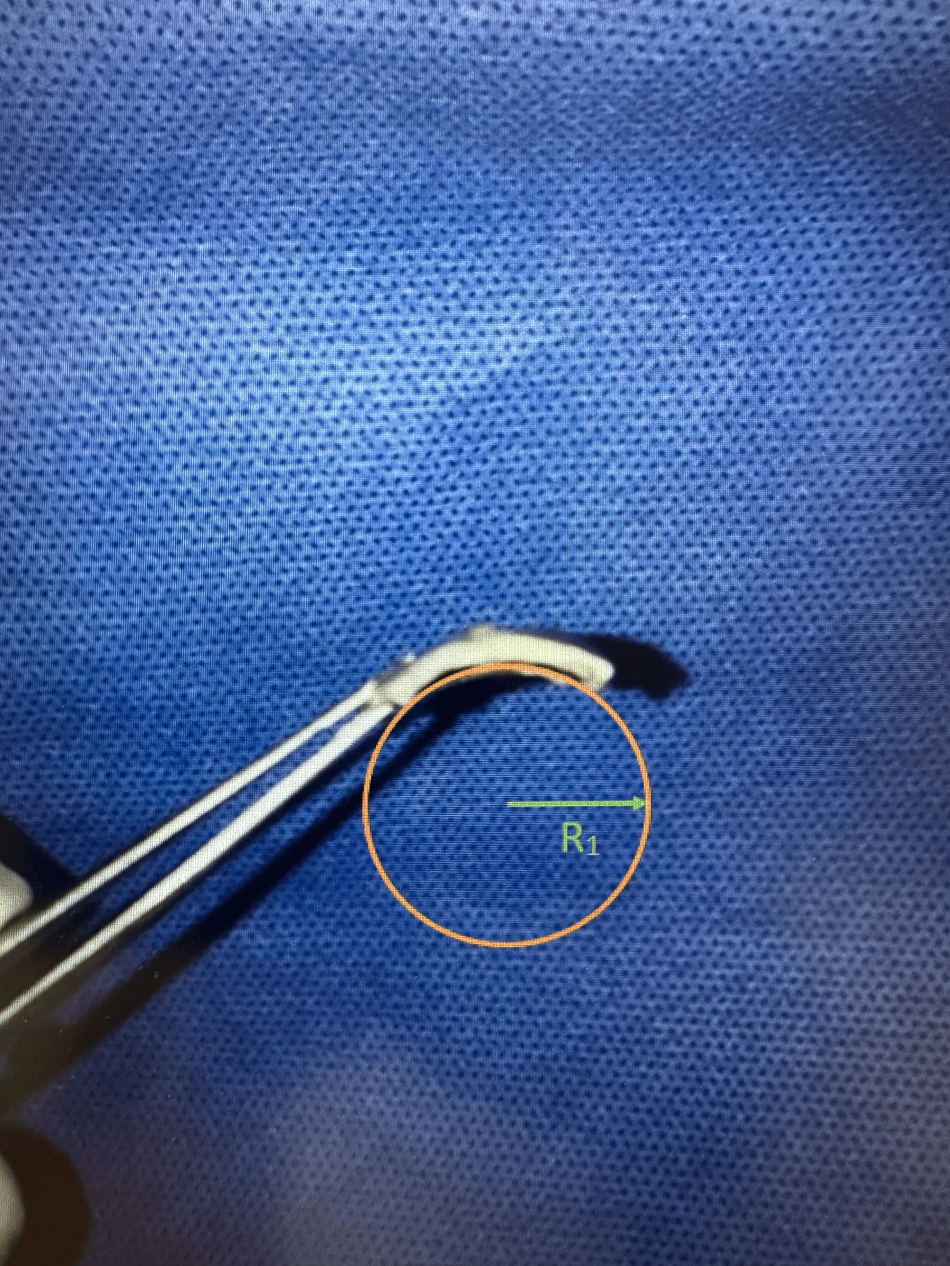
Stiffening effect of porcine sclera after cross-linking with preserved curvature in the treated sclera.

**Figure 4:**
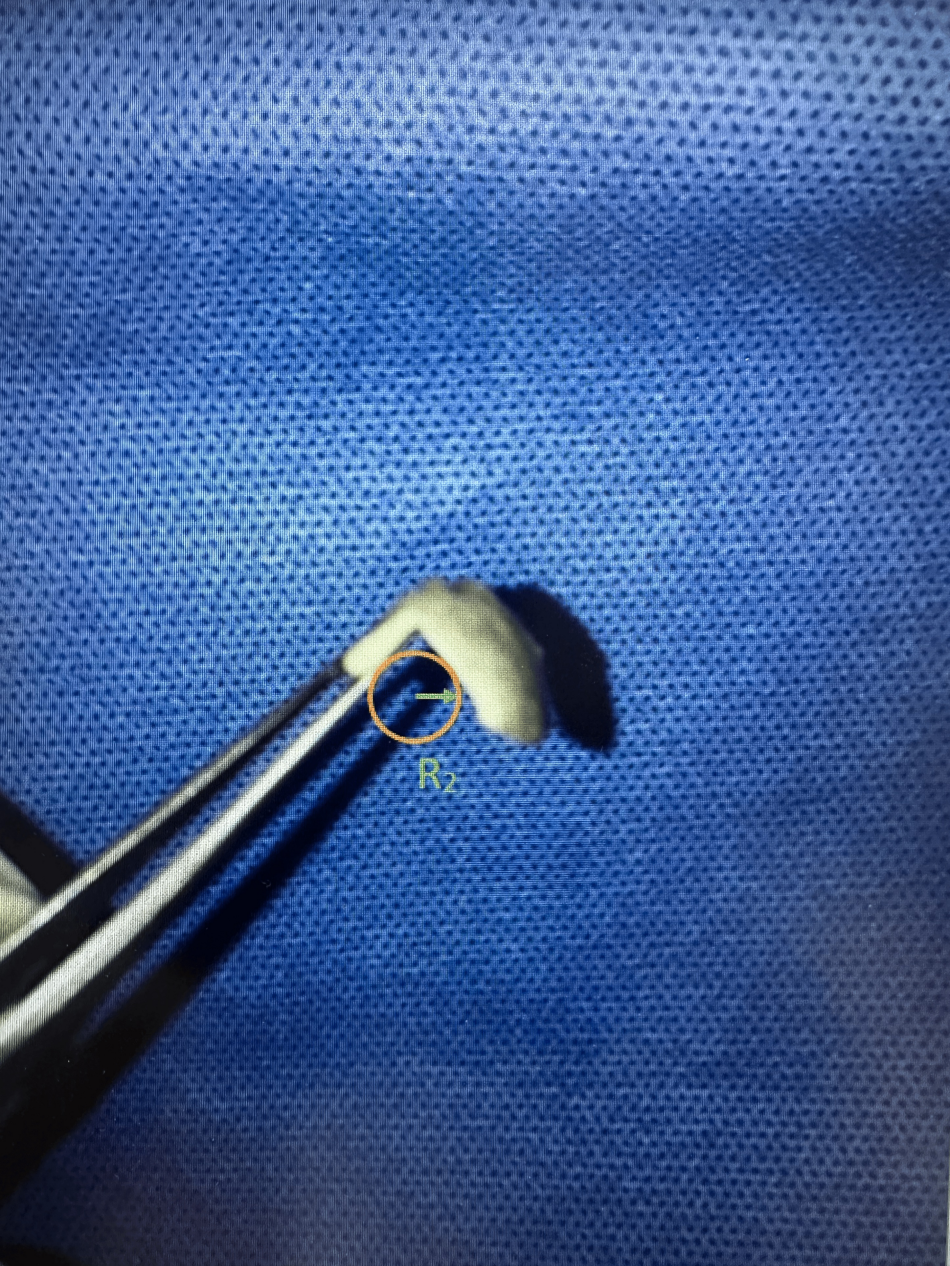
Massive drop of radius of curvature in the untreated control sclera.

The curvature of the scleral strips of both the SXL and control groups was detected using high-resolution photographs. From the captured data, a circle with a known radius of curvature (R) was fitted. The comparison of R between the groups showed that R2 of the control group was significantly dropped compared to R1 of the SXL group (R1>>R2). As curvature is inversely proportional to the radius of curvature, the SXL group showed a significantly stiffer curvature than the control group (Figures 3, 4).

In the control group, the mean IOP observed entails gradual, statistically significant increases for higher volumes. Specifically, the mean IOP at 0 μL equals 10 mmHg (SD = 0), while at a volume of 50 μL, the mean IOP equals 15.18 mmHg (SD = 2.463). The observed values increase as volume increases and at 100 μL the mean IOP equals 19.70 mmHg (SD = 3.332); at 150 μL the mean IOP equals 24.59 mmHg (SD = 2.175); and at 200 μL the mean IOP equals 33.83 mmHg (SD = 4.060). The differences were statistically significant with p-values < .001 in all cases.

Similarly, the observed gradual increases in the SXL group were statistically significant in all cases with p < 0.001 in all cases except for the comparison of volume 0 measurements to volume 50 μL, where the p-value equaled 0.003. Specifically in the SXL group, the mean IOP at 0 μL equals 10.00 mmHg (SD = 0.000), the mean IOP at 50 μL equals 13.31 mmHg (SD = 2.011), the mean IOP at 100 μL equals 17.74 mmHg (SD = 3.346), the mean IOP at 150 μL equals 23.56 mmHg (SD = 2.797), and the mean IOP at 200 μL equals 32.06 mmHg (SD = 3.078).

Table 1 highlights significant increases in IOP with increasing volumes in both groups, while no statistically significant differences were observed between the two groups. Specifically, apart from the volume 0 μL that are absolutely equal, the estimated p-value for the two group differences at volume 50 μL was 0.438, at volume 100 μL was 0.375, at volume 150 μL was 0.968, and at volume 200 μL was 0.526 (Table 1).

**Table 1:** Comparison of intraocular pressure (IOP) values at varying infusion volumes between control and SXL-treated groups. Explanation of acronyms and abbreviations. SD: Standard Deviation, IOP: Intraocular pressure, M (IOP): Mean intraocular pressure, N: number of eyes, Control: Control group, SXL: SXL group, P: p-value

	Control			SXL			
Volume	M (IOP)	SD	N	M (IOP)	SD	N	P
0	10.00	.000	20	10.00	.000	20	1.000
50	15.18	2.463	20	13.31	2.011	20	0.438
100	19.70	3.332	20	17.74	3.346	20	0.375
150	24.59	2.175	20	23.56	2.797	20	0.968
200	33.83	4.060	20	32.06	3.078	20	0.526

The analysis regarding ocular rigidity indicated significantly higher scores in the control group (K50=0.00812, SD = 0.03) compared to the SXL group (K50=0.00552, SD = 0.027) t=2.844; p=0.007. The difference regards measures of volume 0 to 50 μL, while all other rigidity measures were found to be non-significant (Table 2).

**Table 2:** Differences in ocular rigidity between the control and SXL-treated groups. K50: Coefficient of ocular rigidity for Volume 0-50, K100: Coefficient of ocular rigidity for Volume 50-100, K150: Coefficient of ocular rigidity for Volume 100-150, K200: Coefficient of ocular rigidity for Volume 150-200, K: Coefficient of ocular rigidity for Volume 0-200, Control: Control group, SXL: SXL group, N: number of eyes, M (Ocular Rigidity): Mean ocular rigidity, SD: Standard Deviation, t: t-value, p: p-value

	Sclera	N	M (Ocular Rigidity)	SD	t	p-value
K50	Control	20	.00812	.0030	2.844	.007
	SXL	20	.00552	.0027		
K100	Control	20	.00518	.0016	-0.745	.461
	SXL	20	.00565	.0023		
K150	Control	20	.00462	.0030	-1.273	.211
	SXL	20	.00582	.0030		
K200	Control	20	.00631	.0023	0.135	.893
	SXL	20	.00622	.0022		
K	Control	20	.00606	.0006	1.463	.152
	SXL	20	.00580	.0005		

## Discussion

Our study investigated the effects of scleral stiffening induced by riboflavin-UVA scleral cross-linking (SXL) on the relationship between intraocular pressure (IOP) and ocular volume. A methodology closely resembling in vivo conditions was employed to assess changes in ocular rigidity. Our results showed that under physiological conditions, the crosslinking effect in the whole-eye model was comparable between SXL-treated and control globes. Notably, stiffening of the scleral did not significantly alter the pattern of IOP changes in response to variations in ocular volume.

Although it is still a laboratory technique, scleral crosslinking using riboflavin and UVA has emerged as a potential novel strategy in the management of several scleral disorders. Wollensak et al. developed in vitro methods of collagen crosslinking the sclera to increase its biomechanical strength. The concept was based on the observation that artificial corneal stiffening was currently used as a clinical treatment for keratoconus, suggesting that the eye could tolerate local modulation of the stiffness of its collagenous tissues. They used stress-strain measurements to conclude that, at 8% strain, a statistically significant increase in scleral stiffness was found after crosslinking with riboflavin-UVA, glyceraldehyde, and glutaraldehyde in human and porcine scleral strips [8]. Using the same measurement methods to estimate the biomechanical properties of the rabbit sclera following the in vivo cross-linking treatment, they proved that scleral collagen cross-linking with riboflavin-UVA is very effective in increasing the scleral mechanical strength; however, serious side effects were observed in the outer retina [9]. To avoid the serious side effects, they reduced the surface irradiance to 3mW/cm^2^ instead of 4,2mW/ cm^2^ in rabbit models and proved that this method is very effective and constant over a time interval of up to 8 months in increasing the scleral biomechanical strength [10]. Wang M. et al. conducted a study on 15 donor human eyes to assess the biomechanical effects of scleral cross-linking (SXL) by comparing various riboflavin instillation techniques and different cross-linked regions (equatorial and posterior scleral). Their findings indicated that both the equatorial and posterior scleral of human eyes could be effectively strengthened through SXL with riboflavin and ultraviolet A (UV-A) irradiation. Moreover, a 20-minute riboflavin-instilling method was recommended for tissue infiltration before SXL because of its efficacy and potential safety [11]. Similarly, Gawargious et al., operating on 12 human cadaver eyes, concluded that scleral collagen cross-linking by ultraviolet activation of riboflavin differentially increases scleral stiffness more in the equatorial than posterior scleral, whereas Zhang Y et al. showed that riboflavin-UVA SXL can lead to a noticeable increase in the biomechanical stiffness of the sclera, presenting the 40 minutes as the optimum duration of irradiation [13,14]. In our study, we utilized the posterior regions of porcine globes, simulating the area where the posterior staphyloma appears in pathologic myopia, to apply SXL. We embedded them in riboflavin solution for 20 minutes before the irradiation with UVA for 40 minutes. The surface irradiance was 3mW/cm^2^. The macroscopic view of the scleral strips after the SXL application showed that the procedure changed the scleral stiffness.

Our results demonstrated that scleral stiffening did not alter the patterns of IOP increases in response to changes in ocular volume. This finding is not consistent with the stiffened cornea effects on IOP changes. In particular, Jun L. et al., modifying the corneal modulus with glutaraldehyde, showed that stiffened corneas induced substantially higher IOP elevations performing the same amount of intraocular volume change [19].

Asejczyk-Widlicka M. et al. showed that the elasticity moduli (the ratio of stress to volumetric strain) for both cornea and sclera are highly linearly correlated [21]. Since the circumferential stress of the cornea and sclera increases linearly with incremental increases in volume, the difference in response is probably related to the much larger variation in scleral thickness than corneal thickness. In addition, it was observed that as the volume increased, the elastic modulus of the scleral ranged from 3 to 3.5 times greater than that of the cornea. These values were consistent with those found in human eyes for a similar range of applied pressures and were higher than previously reported values (1.5-2.5 times) derived from tonometric measurements [22]. Furthermore, the study highlighted that, with a gradual increase in intraocular pressure (IOP) between 12 mmHg and 25 mmHg, there was no statistically significant change in the average corneal curvature, whereas the scleral curvature showed a linear increase (R² = 0.96) at a rate of 0.07 mm/mmHg. This suggests that the cornea is more resilient to changes in IOP compared to the sclera.

Additionally, the ocular rigidity did not change significantly after SXL. The coefficient of ocular rigidity, calculated according to the original definition of Friedenwald, was found to be 0.0060 μL^-1^ for the control group and 0.0058 μL^-1^ for the SXL group (p=0.152). In lower volumes, the ocular rigidity coefficient (K50) was significantly larger in control eyes than in the SXL group. In higher volumes, the ocular rigidity of the control eyes decreased, and it did not show a significant difference between the groups. In similar studies of enucleated human eyes, Prijot noted a decrease in the coefficient of ocular rigidity of approximately 30% when the intraocular pressure increased from 15 to 55 mmHg. Gloster and Perkins also found that the coefficient of ocular rigidity decreased with pressure [23-26]. Interestingly, in our study, the ocular rigidity coefficient of post-SXL eyes did not show a significant decrease or change with an increase in intraocular pressure.

Finally, increased scleral stiffness might not predict higher ocular rigidity and greater IOP elevations in globes for the volume changes applied in our study. This ascertainment could render the SXL procedure safe against the damaging effects of IOP fluctuations daily. Future studies will examine the safety and efficacy of in vivo SXL to ensure the stiffening effect is maintained in longitudinal studies and, more importantly, to assess whether SXL protects against vision loss in diseases like scleral ectasia and pathological myopia.

The use of a whole-eye methodology that closely resembles in vivo conditions enhances the relevance of the study's findings. By mimicking real-life physiological scenarios, rather than stress-strain measurements, the results are more likely to reflect the true biological effects of scleral stiffening on ocular mechanics, making them more applicable to clinical situations. The fact that stiffening did not dramatically alter this relationship is informative for clinicians seeking to understand the potential effects of SXL on intraocular pressure and eye dynamics. Regarding limitations, the study did not measure important ocular parameters, such as axial length and scleral thickness, which, by definition, are known to influence the overall biomechanical properties of the eye, including rigidity. However, a few studies on this issue did not find any significant dependence of ocular rigidity on either axial length or scleral thickness [27]. Most probably the influence of these factors is small in comparison to other factors: IOP, volume, and material properties. Another limitation concerns the ex vivo nature of this study and the lack of the dynamic physiological processes of living tissues, such as blood flow and cellular responses, that also influence rigidity.

## Conclusions

Cornea cross-linking (CXL) with riboflavin and UVA produces a more stiffened cornea and has already been successfully applied for keratoconus stabilization. Scleral cross-linking (SXL) with UVA and riboflavin has already been used in laboratory studies for scleral stiffness increase as a potential treatment for progressive myopia and scleral ectasia. In this study, we found that increased scleral stiffness induced by SXL does not necessarily lead to higher ocular rigidity or greater intraocular pressure (IOP) elevations in response to volume changes. These findings suggest that SXL is unlikely to aggravate the damaging effects of daily IOP fluctuations. Future research should prioritize evaluating the long-term safety and efficacy of SXL in vivo through longitudinal studies to confirm its sustained biomechanical benefits. Overall, SXL holds promise as a safe and effective therapeutic option for stabilizing scleral biomechanics, preventing vision loss, and managing progressive scleral diseases, pending further validation of its long-term effects.

## References

[1] Flitcroft DI (2012). The complex interactions of retinal, optical and environmental factors in myopia aetiology. Prog Retin Eye Res.

[2] Holden BA (2015). The Charles F. Prentice award lecture 2014: A 50-year research journey: giants and great collaborators. Optom Vis Sci.

[3] Holden BA, Fricke TR, Wilson DA (2016). Global prevalence of myopia and high myopia and temporal trends from 2000 through 2050. Ophthalmology.

[4] Tkatchenko AV, Tkatchenko TV, Guggenheim JA (2015). APLP2 regulates refractive error and myopia development in mice and humans. PLoS Genet.

[5] Morgan IG, He M, Rose KA (2017). Epidemic of pathologic myopia: what can laboratory studies and epidemiology tell us?. Retina.

[6] Backhouse S, Gentle A (2018). Scleral remodeling in myopia and its manipulation: a review of recent advances in scleral strengthening and myopia control. AES Jan.

[7] Jung GB, Lee HJ, Kim JH, Lim JI, Choi S, Jin KH, Park HK (2011). Effect of cross-linking with riboflavin and ultraviolet A on the chemical bonds and ultrastructure of human sclera. J Biomed Opt.

[8] Wollensak G, Spoerl E (2004). Collagen crosslinking of human and porcine sclera. J Cataract Refract Surg.

[9] Wollensak G, Iomdina E, Dittert DD, Salamatina O, Stoltenburg G (2005). Cross-linking of scleral collagen in the rabbit using riboflavin and UVA. Acta Ophthalmol Scand.

[10] Wollensak G, Iomdina E (2009). Long-term biomechanical properties of rabbit sclera after collagen crosslinking using riboflavin and ultraviolet A (UVA). Acta Ophthalmol.

[11] Wang M, Zhang F, Qian X, Zhao X (2012). Regional biomechanical properties of human sclera after cross-linking by riboflavin/ultraviolet A. J Refract Surg.

[12] Liu S, Li S, Wang B (2016). Scleral cross-linking using riboflavin UVA irradiation for the prevention of myopia progression in a guinea pig model: blocked axial extension and altered scleral microstructure. PLoS One.

[13] Gawargious BA, Le A, Lesgart M, Ugradar S, Demer JL (2020). Differential regional stiffening of sclera by collagen cross-linking. Curr Eye Res.

[14] Zhang Y, Zou C, Liu L (2013). Effect of irradiation time on riboflavin-ultraviolet-A collagen crosslinking in rabbit sclera. J Cataract Refract Surg.

[15] Zhang Y, Li Z, Liu L, Han X, Zhao X, Mu G (2014). Comparison of riboflavin/ultraviolet-A cross-linking in porcine, rabbit, and human sclera. Biomed Res Int.

[16] Choi S, Lee SC, Lee HJ, Cheong Y, Jung GB, Jin KH, Park HK (2013). Structural response of human corneal and scleral tissues to collagen cross-linking treatment with riboflavin and ultraviolet A light. Lasers Med Sci.

[17] Sun M, Zhang F, Li Y (2020). Evaluation of the safety and long-term scleral biomechanical stability of UVA cross-linking on scleral collagen in rhesus monkeys. J Refract Surg.

[18] Ou-Yang BW, Sun MS, Wang MM, Zhang FJ (2019). Early changes of ocular biological parameters in rhesus monkeys after scleral cross-linking with riboflavin/ultraviolet-A. J Refract Surg.

[19] Liu J, He X (2009). Corneal stiffness affects IOP elevation during rapid volume change in the eye. Invest Ophthalmol Vis Sci.

[20] Friedenwald JS (1937). Contribution to the theory and practice of tonometry. Am J Ophthalmol.

[21] Asejczyk-Widlicka M, Pierscionek BK (2008). The elasticity and rigidity of the outer coats of the eye. Br J Ophthalmol.

[22] Woo SL, Kobayashi AS, Lawrence C (1972). Nonlinear material properties of intact cornea and sclera. Exp Eye Res.

[23] Pierscionek BK, Asejczyk-Widlicka M, Schachar RA (2007). The effect of changing intraocular pressure on the corneal and scleral curvatures in the fresh porcine eye. Br J Ophthalmol.

[24] Eisenlohr JE, Langham ME, Maumenee AE (1962). Manometric studies of the pressure-volume relationship in living and enucleated eyes of individual human subjects. Br J Ophthalmol.

[25] Prijot E La Rigidité de l'Oeil Humain. Acta Ophthalmologica.

[26] GL J, PE ES (1957). Ocular rigidity and tonometry. Proc R Soc Med.

[27] Perkins ES Ocular volume and ocular rigidity. Exper Eye Res.

